# Fine mapping and gene cloning in the post-NGS era: advances and prospects

**DOI:** 10.1007/s00122-020-03560-w

**Published:** 2020-02-10

**Authors:** Deepa Jaganathan, Abhishek Bohra, Mahendar Thudi, Rajeev K. Varshney

**Affiliations:** 1 Center of Excellence in Genomics and Systems Biology, International Crops Research Institute for the Semi-Arid Tropics (ICRISAT), Patancheru, India; 2 Centre for Plant Molecular Biology and Biotechnology, Tamil Nadu Agricultural University (TNAU), Coimbatore, India; 3 Crop Improvement Division, ICAR-Indian Institute of Pulses Research (IIPR), Kanpur, India

## Abstract

Improvement in traits of agronomic importance is the top breeding priority of crop improvement programs. Majority of these agronomic traits show complex quantitative inheritance. Identification of quantitative trait loci (QTLs) followed by fine mapping QTLs and cloning of candidate genes/QTLs is central to trait analysis. Advances in genomic technologies revolutionized our understanding of genetics of complex traits, and genomic regions associated with traits were employed in marker-assisted breeding or cloning of QTLs/genes. Next-generation sequencing (NGS) technologies have enabled genome-wide methodologies for the development of ultra-high-density genetic linkage maps in different crops, thus allowing placement of candidate loci within few kbs in genomes. In this review, we compare the marker systems used for fine mapping and QTL cloning in the pre- and post-NGS era. We then discuss how different NGS platforms in combination with advanced experimental designs have improved trait analysis and fine mapping. We opine that efficient genotyping/sequencing assays may circumvent the need for cumbersome procedures that were earlier used for fine mapping. A deeper understanding of the trait architectures of agricultural significance will be crucial to accelerate crop improvement.

## Introduction

Genetic improvement in crop plants is a continuous process of developing improved cultivars to meet the ever-increasing human demand for food, nutrition and energy. Natural variations available for important agronomic traits were utilized in crop improvement activities across the world over the century. Mendel’s experiments on genetic inheritance in the nineteenth century constituted the scientific basis for understanding genetics of plant traits and crop improvement through systematic plant breeding. Majority of the agriculturally important traits are complex or quantitative in nature (Abe et al. 2012). Traditionally, these traits were examined using morphological data and statistical analysis based on mean, variance and covariance of relatives. However, these studies achieved limited success because of low level of polymorphism and strong influence by environment. The discovery of molecular marker technology in 1980s made a major breakthrough in understanding the genetics of complex traits. Concurrent refinements in statistical packages enabled construction of genetic linkage maps based on genotypic data for various mapping populations, thus paving the way to discover quantitative trait loci (QTLs) controlling important quantitative traits.

DNA markers associated with the QTL region were used for making rapid and accurate selections and for introgressing traits in many crop species (Kulwal et al. 2012; Varshney 2016). The QTL regions identified by standard mapping procedure often extend to several centiMorgans (cMs) on genetic map (equivalent to several Mbs on physical map) and might contain a large number of genes (Varshney et al. 2014). Therefore, it is very difficult to pinpoint causative locus responsible for a specific trait. Moreover, introgression of such broad QTL regions based on flanking markers may carry undesirable genes (linkage drag), thereby affecting the performance of improved cultivars carrying the introgressed genomic segments. Therefore, genetic resolution of the mapping procedures should be enhanced to allow QTL placement within the shortest possible genomic region (marker interval) using innovative strategies. This process of refining the QTL region is called as fine mapping.

Three factors, viz. population size (Dinka et al. 2007), phenotyping (Cobb et al. 2013) and number of markers, mainly determine the success of QTL dissection, fine mapping and further cloning of the QTLs. Conventional fine mapping process involves screening of a large number of individuals with the DNA markers flanking the target QTLs, followed by phenotyping of the selected recombinant plants and progeny testing. The fine-mapped region obtained through this process is positioned on the physical map, and candidate genes are then identified. In recent years, the discovery of single-nucleotide polymorphism (SNP) markers in combination with evolving sequencing technologies has led a remarkable improvement in fine mapping procedures. SNPs are universal and the most abundant class of genetic variation among the individuals of a given species. High amenability of SNP markers to automation has broken the dominance of medium-throughput simple sequence repeat (SSR) markers that dominated crop research and breeding during the last two decades. Furthermore, massively parallel or high-throughput NGS technologies dramatically reduced per sample genotyping/sequencing cost and increased throughput (Varshney et al. 2009a). As a result, whole-genome sequences are available for majority of the important crop species (Michael and Jackson 2013, https://www.ncbi.nlm.nih.gov/assembly/organism/2759/all/). With the latest SNP genotyping platforms in place, it is now possible to genotype tens of thousands of samples in a short span of time. In this review, we discuss and critically appraise the efforts to fine-map QTLs, cloning QTLs/genes and identification of candidate/causative genes in the pre- and post-NGS era.

### Genotyping tools and approaches for fine mapping in the pre-NGS era

In the early 1990s, DNA-based markers like restriction fragment length polymorphism (RFLP), amplified fragment length polymorphism (AFLP), SSR markers were used for trait dissection. Tomato (*Solanum lycopersicum*) is the first model crop plant species where the use of RFLP markers and QTL identification were reported (Paterson et al. 1988). In the pre-NGS era, SSR markers were most extensively used in genetic and plant breeding studies owing to their several advantages over other marker systems including higher polymorphism rate, genome-wide distribution and amenability to automation (Gupta and Varshney 2000).

**Fig. 1:**
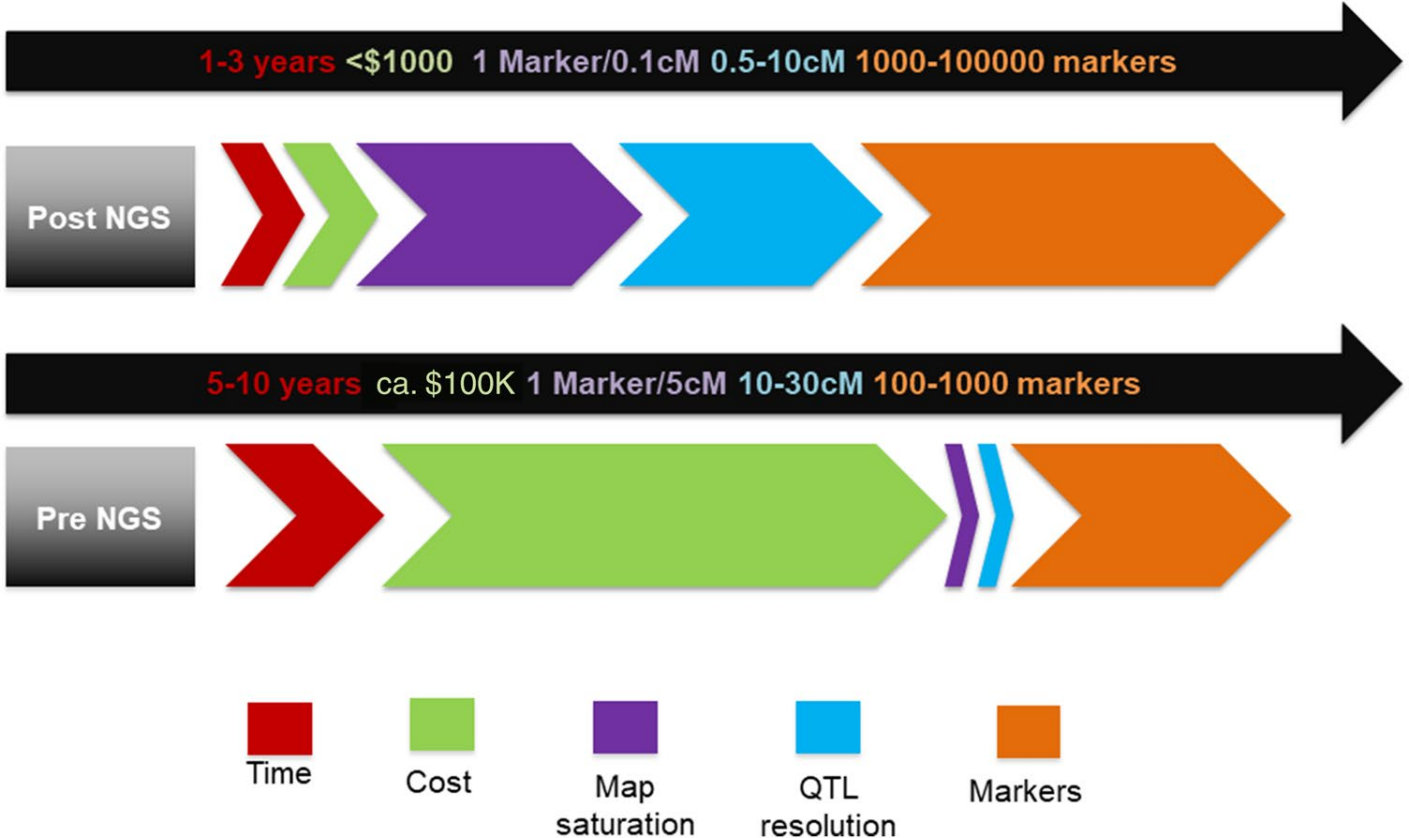
Comparison of marker development, map and QTL resolution during the pre- and post-NGS era. In the pre-NGS era, QTL resolution is low (10–30 cM), while in the post-NGS era the QTL resolution is high (0.5–10 cM)

In the pre-NGS era, QTL cloning involved two broad steps: Firstly, QTL region underlying the trait of interest is identified by using a limited number of DNA markers. Later, the identified QTL region is refined by mapping the QTL-flanking DNA markers onto their physical positions in order to identify respective BAC (bacterial artificial chromosome) clone on the physical map. The clones thus identified were used for sequencing and developing DNA markers within the QTL region (Fig. 1). A limited number of studies have reported fine mapping using SNP markers obtained from BAC/YAC clones in the pre-NGS era (Fridman et al. 2000; Kamolsukyunyong et al. 2001; Wang et al. 2009). Such studies could reach gene level of refinement, demonstrating the potential of SNP markers in refining the broad QTL regions. Although SNPs are more advantageous than other DNA-based makers, lack of high-throughput SNP discovery and genotyping methods in pre- NGS era greatly constrained their widespread utilization for fine mapping studies.

### Genotyping technologies and approaches for fine mapping in the post-NGS era

**Fig. 2:**
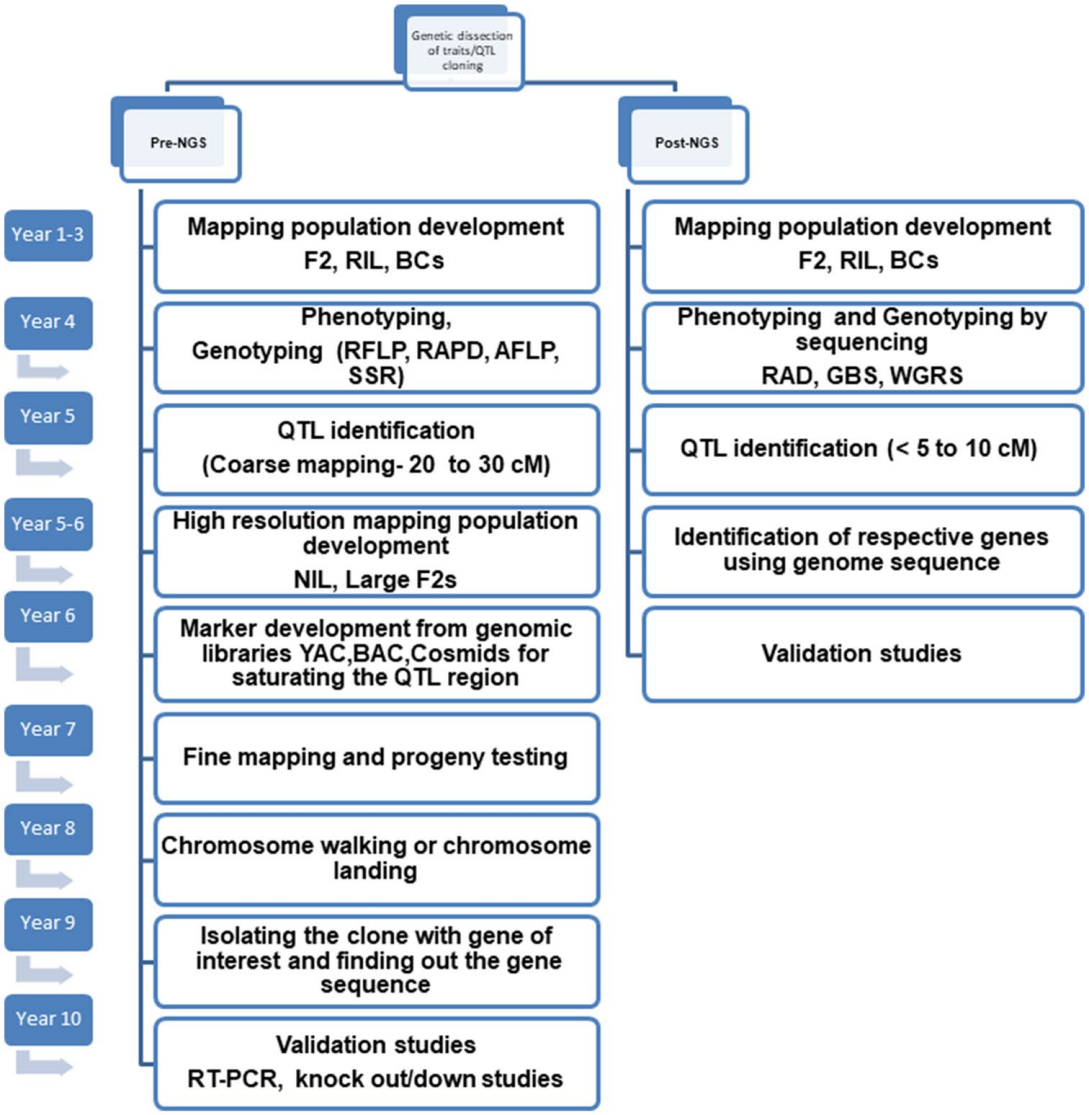
Diagrammatic illustration of duration required for fine mapping during the pre- and post-NGS era. Development of NGS-based markers has nearly reduced half the time span taken for fine mapping using non-NGS-based markers

Increasing adoption of NGS-based assays for population genotyping has facilitated high-density linkage mapping in various crop species (Varshney et al. 2019a). Timeline for fine mapping in both the pre- and post-NGS era is compared in Fig. 2. Most importantly, the NGS technology has allowed parallelization of sequencing process, thereby generating thousands to millions of DNA sequences in a single run and reducing the sequencing cost over 1000-folds since its invention (Park and Kim 2016). New NGS-based protocols have emerged such as reduced representation libraries (RRLs), restriction-site-associated DNA sequencing (RAD), genotyping-by-sequencing (GBS), whole-genome resequencing (WGRS) and skim GBS that are capable of identifying and mapping massive number of SNPs in thousands of samples in one go (Varshney et al. 2019a). A dramatic reduction in sequencing cost has motivated researchers to obtain deeper view of the target genomic region by sequencing entire mapping populations. Additionally, one can generate new markers with lesser efforts and time using the available genome sequence for an identified QTL region using flanking marker information, which was not the case in the pre-NGS era (Fig. 2).

**Fig. 3:**
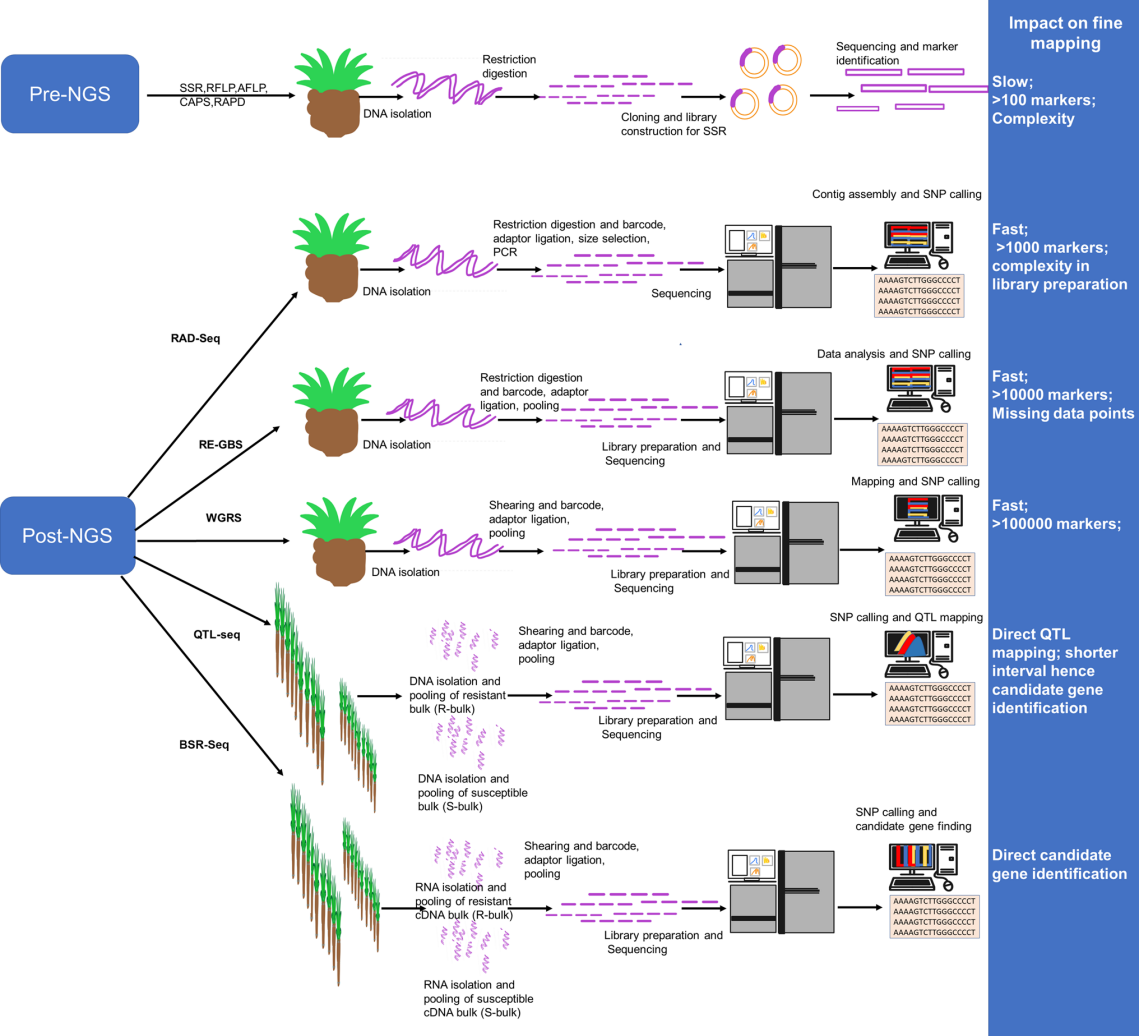
Various techniques followed for fine mapping during the pre- and post-NGS era and their impact on fine mapping

The NGS technologies have offered more benefits to crops that lack whole-genome sequence information, as these technologies generate large-scale DNA markers by sequencing the entire population under study. For example, building a genetic linkage map with moderate density (1000 loci) in the pre-NGS era demanded considerable time and the effort of few technicians in comparison with the current NGS assays that allow highly saturated genetic maps (100,000 loci) to be constructed within few months with modest technical efforts (Yang et al. 2015). Based on the use or non-use of restriction enzymes, the NGS methods can be grouped into two categories: (i) whole-genome sequencing (including WGRS, RNA sequencing, exome capture) that does not employ restriction enzymes, (ii) reduced representation sequencing (such as RAD-seq, GBS, etc.) that uses restriction enzymes for reducing genome complexity (Fig. 3). As these techniques are adequately reviewed elsewhere, they are not discussed in detail in this review (Davey et al. 2011; Garg and Jain 2013; Goodwin et al. 2016).

### Role of NGS in accelerating high-resolution mapping and gene discovery

Several new approaches have led to the fast gene discovery through high-resolution mapping using NGS technologies. We list few important such methods in this section.

*High*-*density SNP arrays for faster mapping* The SNP arrays or high-density genotyping based on resequencing is being used to generate large-scale marker profiles for a number of individuals (Rasheed et al. 2017; Pandey et al. 2017a; Roorkiwal et al. 2018; Saxena et al. 2018). The SNP chip is preferred over other high-density genotyping platforms as the SNP data it generates are less computationally demanding (Yuan et al. 2019). A high-quality SNP chip array, CottonSNP80K, was developed especially for intraspecific genotyping in cotton, and eight SNPs were found to be associated with salt stresses (Cai et al. 2017). In wheat, a high-throughput genotyping array (TaBW280K) containing 280,226 SNPs was developed and deployed for assessing the germplasm diversity as well as high-density linkage mapping (Rimbert et al. 2018). Similarly, SNP chip array containing 6,000 SNPs was developed in castor from WGRS dataset of 14 diverse lines (Senthilvel et al. 2019). This SNP chip array was validated on 314 inbred castor lines and can be applied in genome-wide analysis.

*Bulked segregant RNA Seq (BSR*-*Seq) for gene discovery* Whole transcriptome sequencing of contrasting bulks is referred to as BSR-Seq. The technique is particularly important for crops with large and complex genomes like wheat where resequencing still remains cost-ineffective (Liu et al. 2012a, b). Also, BSR-seq is suitable for fine mapping in crops that still lack a reference genome sequence. Technically similar to QTL-Seq, BSR-Seq relies on sequencing RNAs (cDNA) from extreme bulks for the trait of interest. It facilitates identification of the target region and development of markers near or within the gene of interest. For instance, BSR-seq assigned glossy 3 (*gl3*) gene of maize to ~ 2 Mb region and a single gene myb transcription factor was reported in this region. This gene was reported to control the genes involved in long-chain fatty acid synthesis in maize (Liu et al. 2012a, b). Similarly, BSR-Seq enabled fine mapping of grain protein content (GPC) region in wheat (GPC-B1) to 0.4 cM from previously reported 30 cM. This study pinpointed candidate genes (13–18 genes) for grain protein content in wheat (Trick et al. 2012). In another study, BSR-Seq approach identified marker associated with the *Yr15* gene that imparts resistance to yellow rust in wheat. It facilitated fine mapping of this region to a shortest distance of 0.77 cM. These markers were also utilized to analyze germplasms and can be used in marker-assisted selection (Ramirez-Gonzalez et al. 2015). A more recent study on BSR-Seq allowed cloning of mutant genes in maize that are involved in plant growth via delineation of mapping interval and candidate SNPs from whole-genome sequencing of pooled F_2_ individuals (Klein et al. 2018). Thus, BSR-Seq approach is being widely adopted for rapid discovery of genes and markers linked with the target genes.

*QTL*-*Seq for gene identification* QTL-Seq integrates traditional bulk segregant analysis (BSA) with sequencing methods. This approach has been established as highly efficient for rapid discovery of candidate genes for the trait of interest. By allowing placement of QTL within a smaller genomic segment, it facilitates both detection of QTL and its fine mapping at a stretch. The procedure involves creation of extreme bulks by selecting lines with extreme low and high values of trait from a segregating population. The pools along with one of the parental genotypes are subsequently sequenced using NGS. A reference genome assembly for the parental genotype is developed based on sequencing data. Subsequently, SNP index for each bulk is computed based on the number of reads for a SNP that differs from the SNP in the parent reference assembly. (If 10 reads are having unique nucleotide as in the reference assembly, then SNP index is 0, i.e., 0/10.) Similarly, if all reads have a different nucleotide compared to the reference parent nucleotide, then the SNP index is 1 (10/10). Based on these analyses, Δ-SNP index value of high and low bulks is calculated and the candidate genomic region for the trait of interest is identified. This approach was successfully demonstrated for faster identification of QTLs for blast resistance in rice (*Oryza sativa*) (Takagi et al. 2013a). Similarly, rapid discovery of a major QTL for early flowering was undertaken in cucumber (*Cucumis sativus*) (Lu et al. 2014). In the case of chickpea (*Cicer arietinum*), Singh et al. (2016a) refined a 7 Mb QTL region on linkage group (LG) CaLG04 (previously identified using non-NGS-based markers) to a ~ 1 Mb region for root and seed weight traits in chickpea. Another QTL-seq study in chickpea delineated a 35 kb genomic region on CaLG01 controlling 100-seed weight (Das et al. 2015). In the case of pigeonpea (*Cajanus cajan*), sequencing of resistant and susceptible bulks in combination with WGRS data of four additional genotypes elucidated candidate genes associated with Fusarium wilt and sterility mosaic disease resistance (Singh et al. 2016b). Similarly, QTL-Seq approach was applied to fine-map bacterial wilt resistance genes and develop diagnostic markers for use in breeding in the case of groundnut (Luo et al. 2019a). Adoption of QTL-Seq is increasingly reported for delineating candidate QTLs for both qualitative and quantitative traits (Yang et al. 2017; Li et al. 2018; Zhang et al. 2018; Clevenger et al. 2018; Zhang et al. 2019; Luo et al. 2019b).

#### Mutation mapping techniques for gene identification

*MutMap* The MutMap technique was proposed by Abe et al. (2012) in rice to identify genomic regions governing important agronomic traits. In brief, this technique involves generation of a mutant population using chemical mutagen followed by the selection of line with desirable phenotype in M_2_ or in the subsequent generations. Such selected mutant is crossed with wild-type parent, and the F_1_ is selfed to generate an F_2_ population segregating for the mutant and wild-type phenotypes. DNA samples of F_2_ lines showing mutant phenotype are bulked and subjected to WGRS. The causative SNP for the specific trait is determined based on the SNP index. If all the short reads covering a particular genomic position share a SNP that differs from the reference, the SNP index is defined as 1. The identified genomic regions with the SNP index of 1 are the causal locus underlying the mutant phenotype.

*MutMap+* This technique involves direct sequencing of M3 population instead of backcrossing with wild-type plant; thus, the mutants which are not amenable for crossing in MutMap technique can be utilized for the study. Similar to QTL-seq, mutant and wild-type bulks are prepared using M3 lines and subjected to WGRS. Genomic loci responsible for trait of interest are then inferred with the help of SNP index (Feik et al. 2013).

*MutMap-Gap* MutMap-Gap facilitates identification of the causal SNPs in the genomic regions that are missing from the reference genome sequence. The method involves: (i) identification of the sequences unmapped with the reference genome, (ii) delineation of the candidate genomic segment with MutMap approach, (iii) creation of a de novo assembly of the target region through combining short reads pinpointed in steps i and ii, (iv) alignment of the bulk-sequenced reads (of mutant individuals) to the ‘P + scaffolds’ reference (reference sequence combined with scaffolds obtained in step iv, and finally (v) identification of the causal mutation within the gap region (Takagi et al. 2013b).

*MutChromSeq* Another complexity reduction sequencing approach is mutant chromosome sequencing (MutChromSeq), which involves mutagenesis and screening for mutants followed by chromosome sorting of the selected mutant and sequencing the specific chromosome to identify the causative mutation (Steurnagel et al. 2017). Sánchez-Martín et al. (2016) compared sequence information of multiple independently derived mutant flow-sorted chromosomes which would allow the identification of induced, causal mutations without the need for positional fine mapping in barley and wheat. MutChromSeq can overcome the limitations of RNA Seq (tissue specific, time of sampling and sequencing depth) and exome sequencing (captures  known genes).

### Exome sequencing

Whole-exome sequencing allows us to find out the variations in the protein coding regions and thus enhances the identification of disease-causing mutations in the target gene. The cost-effectiveness of this approach stems from the fact that it involves sequencing of known targeted region instead of the whole genome. It is also extended to capture all the functional region of a genome including noncoding genes and regulatory elements such as promoters and enhancers (Warr et al. 2015). Several targeted genome sequencing techniques are available for faster mapping and gene identification, such as (i) *Resistance gene enrichment sequencing (RenSeq) technology for rapid cloning:* RenSeq is a NBS-LRR gene-specific R gene enrichment method, which allows rapid identification of disease resistance genes by targeted resequencing. This technique includes bait design using known NBS-LRR gene families followed by sequencing of the enriched samples NBS-LRR and genome annotation. As a proof-of-concept study, Jupe et al. (2013) demonstrated its utility in potato (*Solanum tuberosum*) and tomato. In this study, target enrichment library was prepared using 523 NB-LRR-like sequences from potato genome, 57 tomato NB-NRC domains, 9 characterized NB-LRR types from tomato, tobacco (*Nicotiana tabacum*) and pepper (*Capsicum annum*). The enriched samples against genomic DNA of the sequenced *S*. *tuberosum* Group Phureja clone were sequenced, and annotation was carried out. This resulted in successful enrichment of NB-LRRs from 438 to 755. (ii) MutRenSeq: Subsequently, Steuernagel et al. (2016) proposed mutational R gene enrichment sequencing (MutRenSeq) that combines chemical mutagenesis with exome capture and sequencing for rapid cloning of resistance genes such as stem rust resistance genes Sr22 and Sr45 in hexaploid wheat. MutRenSeq significantly reduces the duration of gene cloning process from 5–10 years to 2 years. (iii) SMART-RenSeq: Witek et al. (2016) employed SMART-RenSeq (single-molecule real-time RenSeq) to clone a gene responsible for resistance to *Phytophthora infestans* (Rpi-amr3i) causing late blight disease in potato. (iv) AgRenSeq: In order to develop broad range of disease resistance in domesticated crops, R genes from wild relatives can be utilized. To achieve this, association analysis was combined with RenSeq approach to develop AgRenSeq method (Arora et al. 2019). This technique involves screening of wild plants for variety of diseases and sequencing of the wild plants to look for resistance genes. It was successfully applied in wheat for the rapid identification of resistance genes for stem rust disease. Any crop with diverse germplasm can be subjected to AgRenSeq approach for R gene cloning. Recent examples have shown utility of RenSeq for improving disease resistance in plants, and similar technique for abiotic stress-tolerant gene identification will benefit crops affected with abiotic stress. (v) Targeted chromosome-based cloning (TACCA) via long-range assembly follows an approach similar to MutChromSeq where prior information about the mapped gene (flanking markers of a QTL) and its chromosomal location is used for chromosome sorting and sequencing. Thind et al. (2017) cloned leaf rust resistance gene Lr22a in wheat using this technique. Two SSR markers flanking Lr22a covering 0.48 cM interval on chromosome 2D were mapped previously, the chromosome 2D was sorted followed by sequencing, and the causative genes were identified within four months. These targeted sequencing approaches reduce time and cost in achieving faster gene discovery compared to WGRS if prior knowledge about the gene or loci of interest is available.

### *Gene editing* for candidate gene characterization

Gene editing is a versatile tool identified in this decade for gene characterization and creation of novel alleles. Taking advantage of the sequencing technologies, the candidate genes are identified and validated genome wide, and almost any gene underlying any trait of interest can be dissected. For instance, Lou et al. (2017) reported the role of *OsSAPK2* through creating loss of function mutants by CRISPR/Cas9 approach in rice. This study targeted the third exon of *OsSAPK2* (SNF 1-RELATED PROTEIN KINASE 2) for sgRNA designing. The mutant lines (*sapk2*) were more sensitive to drought and reactive oxygen species (ROS) than wild-type plants. This study suggested that the *OsSAPK2* will be a candidate gene for drought tolerance in rice. Recent reviews highlight increasing contributions of CRISPR/cas9 toward novel gene identification and characterization in various crops (Arora and Narula 2017; Jaganathan et al. 2018; Chen 2019; Varshney et al. 2019b).

### Evolving mapping resources for better resolution of genetic architectures

#### QTL mapping with standard mapping populations

An appropriate mapping population developed from genetically diverse and contrasting parents is a prerequisite of QTL identification (Collard et al. 2005). Various mapping populations such as F_2_, recombinant inbred line (RIL) and double haploid (DH) have been used for QTL identification (Varshney et al. 2009b). Although each population has its own advantages and disadvantages, a RIL population is the most commonly used for QTL mapping studies. Besides, the size of mapping population remains an important factor and depends on a variety of other factors such as type, genetic nature of target trait and access to genotyping and phenotyping facilities; a mapping population comprising 50–250 or more individuals is generally required for preliminary QTL mapping (Collard et al. 2005). A larger mapping population will result in high-resolution mapping of major and minor QTLs. However, the size of mapping population in the pre-NGS era for detecting the QTL was often limited to 200–300 individuals due to lack of high-throughput genotyping methods. Once the QTL for target trait is identified through coarse mapping, populations for fine mapping are developed with large number of progenies (~ 500 to < 10,000) to capture enough recombination to place QTL into a shorter genomic segment (Table 1). A growing body of research suggests NILs as the most preferred population for fine mapping studies as the genetic background is similar across the population except for the target genomic region. Such populations allow the effect of the QTL to be observed accurately, and resolving them into a shorter interval is possible (Fridman et al. 2000; Jander et al. 2002; Uga et al. 2013; Song et al. 2015).

**Table 1:** List of QTL cloning and fine mapping studies reported during the pre-NGS and post-NGS era

Crop	Study	Trait	Primary mapping^a^	Fine mapping	Population Size	Markers used^b^	Refined Size	References
Pre-NGS								
Arabidopsis	QTL cloning	Flowering (FRI and FIC)	M_2_ lines	Test cross lines	4500	SSR	15 kb	Michaels and Amasino (1999)
	QTL cloning	Ascorbate biosynthesis (VTC2)	F_2_	F_2_	3700	InDel	20 kb	Jander et al. (2002)
Rice	QTL cloning	Grain width and weight (GW2)	F_2_	BC_3_F_2_	6013	RFLP	82 kb	Song et al. (2007)
	QTL cloning	Grain number	BIL	NIL + × NIL − _F_2_	13,000	SNP	63 kb	Ashikari et al. (2005)
	QTL cloning	Heading date	RIL	RIL + × P1_BC_2_F_2_	8400	SSR	2 Mb	Xue et al. (2008)
	QTL cloning	Grain weight	F_2_	BC_2_F_2_	674	STS	122 kb	Guo et al. (2009)
	QTL cloning	Heading date (Hd1)	F_2_	BC_3_F_3_	> 9000	RFLP, CAPS	71 kb	Yano et al. (2000)
	QTL cloning	Submergence (Sub1A)	F_3_	F_2_	2950	AFLP	182 kb	Xu et al. (2006)
	QTL cloning	Photoperiodic flowering (Ehd1)	BC_2_F_1_	NILs (BC_6_F_2_)	>2500	RFLP, CAPS	115 kb	Doi et al. (2004)
	QTL cloning	Seed hull color	CSSL	F_2_ (NIL)	3276	SSR, SNP	88 kb	Zhu et al. (2011)
	QTL cloning	Rooting depth	RIL	NIL (BC_3_F_2_)	4560	SSR	60 kb	Uga et al. (2013)
	QTL cloning	Grain weight (OsglHAT1)	BIL	F_3_ (NIL)	3012	SNP	12 kb	Song et al. (2015)
	Fine mapping	Photoperiod sensitivity	BCF_4_F_2_	BC_3_F_3_/BC_4_F_3_	2807	CAPS	264 kb	Takahashi et al. (2001)
	Fine mapping	Stele transversal area	F_3_	BC_2_F_1_ to BC_2_F_4_	8–160	InDel	359 kb	Uga et al. (2011)
Maize	QTL cloning	Plant architecture (tb1)	F_2_	F_1_ and F_2_	26,000 and 42	RFLP	15 kb	Doebley et al. (1997)
	QTL cloning	Leaf angle (ZmCLA4)	F_3_	BC_5_F_2_ (BC_3_F_1_ to BC_5_F_2_)	10,628	SSR	48 kb	Zhang et al. (2014)
	QTL cloning	Glume architecture (Tga1)	F_2_	F_2_	3106	SNP	1042 bp	Wang et al. (2005)
	QTL cloning	Flowering time (vgt1)	NIL	F_2_ (NIL)	4526	AFLP, CAPS, ASPCR	2 kb	Salvi et al. (2007)
	Fine mapping	Maize streak virus resistance	F_3_	F_2_	4725	SNP	762 Mb	Nair et al. (2015)
	Fine mapping	Rough dwarf resistance (qMrdd1)	HIF	BC_1_F_3_	2685	SSR, SNP	12 Mb	Tao et al. (2013)
	QTL cloning	Root initiation (RTCS)	F_2_	F_2_	2000	SSR, CAPS	735 bp	Taramino et al. (2007)
Wheat	QTL cloning	Dormancy	MAGIC	HIF, NIL	5 NIL sets	SNP	3 genes	Barrero et al. (2015)
	Fine mapping	Grain weight	BC_2_F_3_	BC_4_F_2_, BC_4_F_3_	118 and 264	SSR	76 cM	Roder et al. (2008)
Barley	Fine mapping	Frost resistance	DH	F_2_	1849	RFLP, SNP, CAPS	0.81 cM	Francia et al. (2007)
	Fine mapping	Grain Threshability	BC_2_DH	BC_4_F_2_	7000	SSR, SNP	43 cM	Schmalenbach et al. (2011)
	QTL cloning	Boron tolerance (Bot1)	DH lines	F_3_	6720	CAPS	0.05 cM	Sutton et al. (2007)
Soybean	Fine mapping	Seed number and leaflet shape	BC_3_F_2_	BC_3_F_3_	4635	SSR	66 kb	Jeong et al. (2012)
Tomato	QTL cloning	Fruit weight (fw22)	F_2_	F_2_	3472	RFLP, RAPD, CAPS	663 bp	Frary et al. (2000)
	QTL cloning	Sugar content (brix9-2-5)	NIL	F_2_	7000	RFLP, RAPD, CAPS	484 bp	Fridman et al. (2000)
	QTL cloning	Locule number (lc)	F_2_	F_2_	9456	SNP, CAPS	1608 bp	Munos et al. (2011)
	Fine mapping	Tomato yellow leaf curl virus resistance	F_3_	F_4_	11,000	SSR	491 kb	Yang et al. (2014)
	Fine mapping	Fruit mass and Brix	IL	IL	50	RFLP	32 and 12 cM	Eshed and Zamir (1995)
	Fine mapping	Sugar content	IL	F_2_ (NIL)	7000	RFLP, SNP	484 bp	Fridman et al. (2000)
	Fine mapping	Fruit mass	IL	NIL	3472	RFLP, RAPD	150 kb	Alpert and Tanksley (1996)
Post-NGS								
Arabidopsis	Fine mapping	Flowering time	F_2_	NA	192	GBS-SNP	9 kb	Rowan et al. (2015)
	Fine mapping	Rosette leaf number (recq4a)	F_2_	NA	192	GBS-SNP	269 kb	Rowan et al. (2015)
Rice	Fine mapping	Leaf width, aluminum tolerance	RIL	NA	176	GBS-SNP	< 2 Mb	Spindel et al. (2013)
	Fine mapping	Plant height	RIL	NA	150	WGRS-SNP	100 kb	Huang et al. (2009)
	Fine mapping	Tillering and panicle branching	F_2_	NA	1642	SSR, SNP	63 kb	Yu et al. (2017)
	Fine mapping	Brown planthopper (BPH31)	F_2_	NA	27	InDel	475 kb	Prahalada et al. (2017)
	Fine mapping	Grain weight, grain length, grain width	BIL	NA	185	GBS-SNP	32–363 kb	Bhatia et al. (2018)
Maize	Fine mapping	Tassel and ear architecture	F_2_	NA	708	GBS-SNP	08–566 Mb	Chen et al. (2014)
Wheat	Fine mapping	Grain protein content (GPC-B1)	RSL	F_3_	28	SNP	0.45 cM	Trick et al. (2012)
	Fine mapping	Powdery mildew (PmTm4) resistance	F_2_	NA	1499	SNP	066 cM	Xie et al. (2017)
Barley	Fine mapping	Awn length	HIF	NA	927	SNP	< 0.9 cM	Liller et al. (2017)
Sorghum	Fine mapping	Grain weight	F_2_	F_3_	307	SSR	101 kb	Han et al. (2015)
	Fine mapping	Seed dormancy	F_2_	F_3_	80	SSR	96 kb	Li et al. (2016a)
Common bean	Fine mapping	Angular leaf spot resistance (ALS41^GS,UC^)	F_4_, Backcross lines	NA	180	SSR, SNP	418 kb	Keller et al. (2015)
Chickpea	Fine mapping	Ascochyta blight	RIL bulks	NA	20	QTL-Seq SNP	15–64 Mb	Deokar et al. (2019)
Soybean	Fine mapping	Phytophthora resistance	F_2_	F_3_	826	SSR, CAPS, SNP	36 kb, 151 kb	Li et al. (2016b)
	Fine mapping	Root knot nematode resistance	RIL	NA	246	WGRS-SNP	297 kb	Xu et al. (2013)
Tomato	Fine mapping	Fruit shape (fs81)	Backcross lines	NA	3	SNP, dCAPS	303 Mb	Sun et al. (2015)

^a^Abbreviations for primary population used: *CSSLs* chromosome segment substitution lines, *HIFs* heterogenous inbred families, *RSLs* recombinant substitution lines, *MAGIC* multi-parent advanced generation inter-cross, *DH* double haploid, *BILs* backcross inbred lines, *NIL* near-isogenic line, *RIL* recombinant inbred line, *IL* introgression line

^b^Abbreviations for markers used: *RFLP* restriction fragment length polymorphism, *RAPD* random amplified polymorphic DNA, *AFLP* amplified fragment length polymorphism, *SSR* simple sequence repeat, *STS* sequence-tagged site, *SNP* single-nucleotide polymorphism, *CAPS* cleaved amplified polymorphic sequence, *dCAPS* derived cleaved amplified polymorphic sequences, *InDel* insertion and deletion, *ASPCR* allele-specific polymorphic chain reaction, *GBS-SNP* SNPs derived from genotyping by sequencing, *WGRS-SNP* whole-genome resequencing, *QTL-Seq* QTL-Seq analysis

Emergence of high-throughput genotyping platforms in the post-NGS era has dramatically transformed the methods employed previously for fine mapping of a candidate genomic segment. Genome-wide SNP markers are now available even for the crops that were earlier known as orphan crops (Varshney et al. 2013a; Bohra and Singh 2015; Varshney et al. 2019a). Therefore, use of genome-wide methodologies is widespread for RIL or F_2_ populations for fine genetic dissection of QTL regions, which was otherwise not possible during the pre-NGS era. Recently, several studies have reported fine mapping and identification of genes from RIL, F_2_ populations (Qi et al. 2014; Wang et al. 2018).

### Innovative experimental designs for enhanced gene discovery

The major drawback of the biparental QTL analysis is that the QTLs are often placed to large chromosomal regions as the inference is derived from limited recombinational events. Further, limited genetic diversity and inadequate polymorphic markers hamper the level of precision with which QTL could be placed in the genome. Later, GWAS was widely used to overcome these issues as it permits consideration of broad genetic diversity for trait mapping. In recent times, experimental populations based on multiple founders have gained widespread attention to accelerate QTL mapping and gene discovery (Bohra 2013). These multi-parental populations offer balanced population structure over GWAS and allow profuse recombination as compared to traditional biparental populations (Bazakos et al. 2017; Wallace et al. 2018). These community genetic resources include multi-parent advanced generation inter-cross (MAGIC) and nested association mapping population (NAM).

MAGIC population is developed by crossing multiple founders through two-way, four-way and eight-way crossing, leading to attainment of a fully inbred recombinant population. The major advantages of MAGIC population include increased recombination, improved mapping resolution and greater allelic diversity (Cavanagh et al. 2008). MAGIC design was successfully implemented in plants for fine genetic mapping including Arabidopsis (Kover et al. 2009), wheat (Huang et al. 2012), rice (Bandillo et al. 2013), chickpea (Gaur et al. 2012), tomato (Pascual et al. 2015), cowpea (Huynh et al. 2018) and so forth. The current status of MAGIC populations in major crops along with the unique opportunities and challenges offered by such mapping resources has been thoroughly discussed elsewhere (Huang et al. 2015).

Another multi-parent mating design NAM involves crossing one common reference genotype with diverse founders to generate a series of “interconnected” segregating inbred families. Like MAGIC, NAM offers the advantages of both linkage analysis and association mapping approaches while overcoming the shortcomings of both approaches. The utility of NAM design for QTL mapping is well established in maize (Yu et al. 2008), and the design has been extended to other crops like wheat (Bajgain et al. 2016), sorghum (Bouchet et al. 2017), barley (Nice et al. 2017), oilseed rape (Hu et al. 2018) and soybean (Song et al. 2017; https://www.soybase.org/SoyNAM/).

### Fine mapping and QTL cloning in the pre- and post-NGS era—notable examples

Recent advances in molecular biology, biotechnology and genomics have facilitated the cloning of QTLs in crop plants. Technological advances and the efforts toward QTL cloning (see Salvi and Tuberosa 2005; Price 2006) and beyond cloning (Anderson and Mitchell-Olds 2010) were critically appraised recently. We summarize fine mapping and cloning procedures followed during the pre- and post-NGS era in model crops like Arabidopsis and tomato followed by major cereals and legumes with few case studies.

### Model plants/crops

*Arabidopsis* Arabidopsis (*Arabidopsis thaliana*) is the first plant genome sequenced, and the availability of the genome sequence of Arabidopsis has brought tremendous changes to the methods of fine mapping and cloning (Lukowitz et al. 2000). In the early 1990s, development of a genetic linkage map with 50 markers required great efforts (Bell and Ecker 1994). More than 4000 BC_1_F_1_ plants were assayed by Michaels and Amasino (1999) for positional cloning of flowering genes (*FLC* and *FRI*). Recombinants were identified using two flanking SSR markers. The marker numbers were increased by four yeast artificial chromosome (YAC) clones from this region. The cleaved amplified polymorphic sequence (CAPS) markers were generated, and further progeny testing delimited the FLC region to a 620 kb interval. Further, analysis of BAC clones in this region delineated a 10–20 kb region with three genes, among which MADS box transcription factor was found to play an important role in flowering in Arabidopsis. This research took almost 5 years from coarse mapping to positional cloning of the flowering gene. The availability of genome sequence of Arabidopsis provided ample marker resources for fine mapping and QTL cloning for the trait of interest (Jander et al. 2002). For instance, VTC2 gene responsible for ascorbic acid deficiency was fine-mapped using the DNA markers from Cereon Genomics within a time span of 2 years.

The key genomic regions associated with flowering time and rosette leaf number were identified using GBS-SNP-based examination of genome-wide crossover. This study identified recombination break points, and QTL analysis using a saturated linkage map determined the location of genes for flowering time and rosette leaf number within 9 and 26.9 kb, respectively. Genome-wide analysis with 215 K SNPs had uncovered candidate genes for time-dependent drought QTLs (Bac-Molenaar et al. 2016). As envisaged by Huang et al. (2009), enhanced precision in the identification of crossovers enabled by new genome-wide genotyping technologies will make current methods of QTL discovery and fine mapping faster, accurate and cost-efficient.

*Tomato* Tomato is one of the earliest targeted crop species for QTL studies, for which molecular markers were available in 1980s itself. The marker repertoire was enhanced from few RFLP markers to millions of SNP in the due course with the release of whole-genome sequence in 2012 (Tomato Genome Consortium 2012). Genomic resources including genome sequences, genome maps, QTLs and gene expression atlas are available in SGN (SOL Genomics Network) database; this provides a complete information of tomato and other crops that belong to *Solanaceae* (Mueller et al. 2005). In 1993, *pto* gene conferring resistance to *P. syringae pv. tomato* was cloned using a high-density map with RFLPs and a tomato YAC library (Martin et al. 1993). The *Pto* gene was genetically mapped using 251 F_2_ progenies; later, the cloned segment/gene was confirmed by analyzing a total of 1300 F_2_ plants, F_3_ families and 50 cultivars using markers spanning the identified YAC segment. This is the first report on map-based cloning of disease-resistant gene in plants. Cloning of fruit weight QTLs in tomato using map-based cloning was successfully performed, and progeny testing involved RFLP assay on a total of 3472 F_2_ plants with markers derived from a YAC contig (Alpert and Tanksley 1996). Later, Frary et al. (2000) screened this YAC contig with a cDNA library followed by cosmid library of *L. pennellii* (small fruited genotype), which identified a candidate gene ORFX, and the underlying mechanism was elucidated with complementation test of this gene. In another study, 7000 F_2_ lines were used for fine mapping of sugar content in tomato (Fridman et al. 2000). Using RFLP markers derived from BAC sequence, a shortest interval of 484 bp of an *invertase* gene was identified by progeny testing. However, the scenario has improved due to the availability of tomato genome sequence (Tomato genome consortium, 2012) and new sequencing techniques which allowed to precisely identify few candidate genes from a large set of genes. For instance, Sun et al. (2015) reported 12 candidate genes controlling tomato fruit shape and other morphological characters from a set of 122 annotated genes in 3.03 Mb region through RNA seq technique (Table 1).

*Cereals* Among cereals, extensive studies on fine mapping and QTL cloning have been conducted in rice, wheat, maize, barley, etc. The reference genomes have become available for almost all major cereal crops such as rice (IRGSP 2005), wheat (Choulet et al. 2010), maize (Schnable et al. 2009), sorghum (Paterson et al. 2009), barley (IBGSC 2012), pearl millet (Varshney et al. 2017). Availability of these reference genomes has greatly facilitated fine mapping and QTL cloning studies on various agronomic traits including response to biotic and abiotic stresses. Few of these genetic studies are listed in Table 1.

*Rice* The first success story of gene cloning in rice appeared even before the availability of genome sequence information of rice. For instance, the gene *Xa21* (identified from *O. longistaminata* (Khush et al. 1990)) was cloned using map-based cloning approach. The *Hd1* locus responsible for photoperiod sensitivity was cloned using a map-based cloning approach on a large BC_3_F_3_ population (Yano et al. 2000). Similarly, analysis of 2807 BC_3_F_4_ plants led to mapping of heading QTL, *Hd6* to a 26.4 kb region and complementation test confirmed its role in late heading in rice (Takahashi et al. 2001). In the post-NGS era, whole-genome resequencing of populations has been adopted in rice for quick identification of candidate genes. Several analytical frameworks were developed to tackle the deluge of the sequence information generated from such whole-genome sequencing projects. One such promising method was proposed by Huang et al. (2009), known as sliding window approach to identify recombination break points using low-coverage WGRS of 150 recombinant inbreds. The interval between two recombination break points is known as bin that served as markers for linkage map construction. This approach has accurately mapped the semi-dwarf gene “*sd1*” on chromosome 1 of rice genome. Another domestication-related gene required for red pericarp *(Rc)* was also cloned (Sweeney et al. 2006). cDNA cloning was also adopted to clone a novel bacterial blight resistance-related gene ME137 from *O. meyeriana* (He et al. 2013). Guo and colleagues cloned the qSD7-1 dormancy QTL underlying gene Os07g11020, which is annotated as a transcription factor and is the same as the red pericarp color gene Rc from wild rice (*O. rufipogan*) (https://portal.nifa.usda.gov/web/crisprojectpages/0214099-characterization-of-the-qsd7-1-seed-dormancy-gene-for-allelic-differentiation-and-regulatory-mechanism-in-isogenic-background-of-rice.html). Using map-based cloning approach, Dai et al. (2012) identified a major quantitative trait locus (QTL) LHD1 (late heading date 1), an allele of DTH8/Ghd8, which controls the late heading date of wild rice and encodes a putative HAP3/NF-YB/CBF-A subunit of the CCAAT-box-binding transcription factor. In another study, using map-based cloning approach, Wang et al. (2015) demonstrated map-based cloning of BPH29 gene, a B3 domain-containing recessive gene conferring brown planthopper resistance in rice. This study used an *Indica* rice introgression line RBPH54, derived from wild rice *O. rufipogon* with sustainable resistance to BPH. Cloning of *An*-*2* gene that encodes the Lonely Guy-like protein 6 (*OsLOGL6*) into *O.* *sativa* ssp *indica* cv GuangLuAi4 (*GLA4*) demonstrated to have a large impact on reducing awn length and increasing tiller and grain numbers in domesticated rice (Gu et al. 2015).

With whole-genome sequence information and NGS protocols in place, several studies have reported characterization of important traits in rice including tillering traits (Yu et al. 2017), disease resistance (Kim et al. 2015; Prahalada et al. 2017), seed longevity (Sasaki et al. 2015), etc. McCouch et al. (2016) launched an open-access high-resolution platform which contains collection of diverse germplasm, high-density SNP marker data and bioinformatics tools for facilitating genome-wide association mapping in rice. For instance, GBS approach has been utilized for QTL identification using nearly 3000 SNPs on backcross inbred lines (BILs) for grain weight and grain length (Bhatia et al. 2018).

*Wheat* Fine genetic mapping of *fusarium* head blight (FHB) resistance QTL to short interval of 1.2 cM was reported in wheat (Liu et al. 2006). Recombinants were selected using SSR and STS markers from a large mapping population with 3156 lines derived from an F_7_ line (RI 63). This study also highlighted micro-collinearity among wheat, rice and barley with respect to the genomic region controlling FHB resistance. Similarly, positional cloning of *VRN1* gene for vernalization in wheat employed a large population for progeny testing (3095 F_2_ plants) and comparative physical maps of rice and sorghum for collinearity analysis of VRN1 region (Yan et al. 2003). BAC contigs and bin-mapped markers from genetic map (ESTs, SSR and RFLP) were used followed by newly developed STS and CAPS markers to fine-map greenbug aphid resistance gene *Gb3* using F_2:3_ population (Azhaguvel et al. 2012). This marker enrichment allowed mapping of *Gb3* gene to a short interval of 1.1 cM of wheat chromosome arm 7DL. Using BSR-Seq, a set of 13–18 genes in syntenic cereal genomes for grain protein content (Trick et al. 2012). Similarly, powdery mildew resistance gene PmTm4 was fine-mapped into 0.66 cM interval using comparative genomics approaches on large F_2_ population (Xie et al. 2017). Furthermore, high-density linkage mapping based on NGS-derived markers has enabled fine mapping of major traits like fungal resistance (Cockram et al. 2015), powdery mildew resistance (Liang et al. 2016), awn shape and length (Yoshioka et al. 2017), flag leaf traits (Hussain et al. 2017) and stripe rust resistance (Ma et al. 2019).

*Maize* Plant architecture is an important phenomenon to study, especially in maize, understanding the genetic control of ear and tassel is important due to their role in grain yield. Doebley et al. (1997) cloned the *teosinte branched 1* (*tb 1*) gene which explains the evolutionary changes during maize domestication. This gene was found to play an important role in repressing the growth of axillary organ and enable the formation of female inflorescences. Initially, QTL for *tb1* was mapped on F_2_ population; further, the QTL was introgressed into different genetic backgrounds to validate the QTL and complementation test has confirmed the role of *tb1* in maize architecture. A similar analysis of fruit case/ear structure on 3106 F_2_ plants delimited a single gene, *teosinte glume architecture* (*tga1*) into 1024 bp controlling ear structure from a wild teosinte to domesticated maize (Wang et al. 2005). Salvi et al. (2007) have cloned a major flowering-time quantitative trait locus, vegetative to generative transition 1 (*Vgt1*) in maize. The cloned region was confined to ~ 2 kb noncoding region positioned 70 kb upstream from Ap2-like transcription factor. A large F_2_ population comprising 10,000 lines derived from N28 × NIL C22-4 was used for the QTL cloning study. NIL C22-4 was obtained through the twenty cycles of straight backcrossing of N28 and Gaspé Flint (an early flowering variety). Tassel and ear architecture were dissected using an early-generation population genotyped with low-coverage GBS assay (Chen et al. 2014). This study reports candidate genes involved in tassel structure in addition to confirming several reported QTLs in the shortest physical interval with less time, cost and effort. Recently, BSR-Seq was applied in maize to understand herbicide resistance mechanism, and cytochrome *P450* gene (CYP81A9) was identified to be the candidate gene of *Nss* associated with nicosulfuron sensitivity in maize (Liu et al. 2019). Applying combination of approaches like linkage mapping and genome-wide association studies has shown its efficacy in narrowing down of the target regions in a short span of time.

*Barley* In the pre-NGS era, the best approach for mapping traits in crops with limited genomic resources relied on exploiting the syntenic relationships with the model crops that have whole-genome sequence information. Hinze et al. (1991) mapped the resistance loci *mlo* on chromosome 4 (2.7 cM) for powdery mildew resistance using RFLP markers on backcross lines. High-resolution mapping of Rym4/Rym5 locus conferring resistance to the barley yellow mosaic virus complex (BaMMV, BaYMV and BaYMV-2) were reported by Pellio et al. (2005). Two high-resolution mapping populations of 1040 F_2_ and 3884 F_2_ lines were developed for mapping rym4 and rym5, respectively.  Combinations of markers including RAPD, AFLP, SSR and CAPS were employed for marker saturation and screening; further, closely linked markers were converted to STS markers. The homozygous recombinants were characterized for disease resistance. This study delimited the Rym4/Rym5 locus into less than 2 cM on genetic map and paved a way for positional cloning. Boron tolerance (*Bot1*) gene was cloned using DH lines and a large mapping population comprising 6720 lines (Sutton et al. 2007). *Bot1* was reported to be the responsible gene for boron tolerance by controlling the net entry of boron into the roots and the disposal of boron from leaves in African barley landrace Sahara 3771. Fine mapping of a semi-dwarfing gene *sdw3* to 0.04 cM was achieved using synteny between barley and other cereal genomes such as rice, sorghum and brassica (Vu et al. 2010). Crops with complex genomes like barley and wheat have been greatly benefitted by the NGS-based protocols like GBS (Poland and Rife 2012). GBS analysis was used to map plant height QTL on RIL population (Liu et al. 2014). Using barley genome assembly, the genes located within the QTL region were identified by mapping the flanking markers on the genome. Similarly, Liller et al. (2017) have fine-mapped a QTL (AL7.1) for awn length to < 0.9 cM on NILs using SNP markers derived from barley consensus map. Barley genome has been sequenced very recently (Mascher et al. 2017) and being explored for fine trait mapping. BSA-Seq analysis suggested nine confident genes resulting from fine mapping of the locus *Rha2* for cereal cyst nematode (CNN) in barley (Van Gansbeke et al. 2019). A seminal paper by Pourkheirandish et al. (2015) in barley reported fine mapping of *btr1* and *btr2* genes to genomic intervals of 1.2 kb and 4.9 kb, respectively, on chromosome 3H by analyzing more than 10,000 F_2_ individuals. The genetic complementation tests validating the identities of *btr1* and *btr2* as ORF 1 and ORF 3, respectively, elucidated key changes occurred during domestication in barley in terms of rachis brittleness and seed dispersal system.

*Sorghum* Several studies have reported QTLs in sorghum for abiotic and biotic stress tolerance and other agronomically important traits; however, only a few of these could reach the level of fine mapping and QTL cloning. In recent years, researchers have increasingly adopted WGRS for building high-density genome maps that serve as foundation to locate QTLs with greater precision and accuracy in sorghum (Zou et al. 2012; Hilley et al. 2017). Fine structure of sorghum aluminum tolerance locus *Altsb* was elucidated following association mapping in a panel of 254 accessions. This study accentuates genetic manipulation of a precisely mapped 6 kb genomic region to confer aluminum tolerance in molecular breeding (Caniato et al. 2014). Fine mapping of shoot fly resistance and stay-green mechanism of terminal drought tolerance-related traits on chromosome SBI-10 was successfully achieved using GBS-SNP-based high-density linkage map on high-resolution fine mapping cross (Kiranmayee 2016). Genome sequencing of sorghum has paved the way for developing molecular markers linked to specific traits by extracting the DNA sequence for the region of interest. For instance, Han et al. (2015) delimited the *qGW1* region to 101 kb region for grain weight in sorghum using SSR markers derived from whole-genome sequence. Similarly, by using SSRs from genome sequence, Li et al. (2016a) mapped *qDor7* QTL for seed dormancy trait onto a genomic region spanning 96 kb with 16 candidate genes. Following linkage mapping and GWAS in two RILs and a diverse panel with GBS, Boyels et al. (2017) found genomic regions controlling grain quality traits. A more recent GWA study in sorghum using GBS-SNPs revealed resistance genes for grain mold fungal disease resistance (Nida et al. 2019).

### Legumes

Legumes have lagged far behind those of cereals with respect to fine mapping and QTL cloning. However, fine mapping studies have gained momentum in recent years and the progress is likely to accelerate following the availability of whole-genome sequences of these legume species including soybean (Schmutz et al. 2010), pigeonpea (Varshney et al. 2012), chickpea (Varshney et al. 2013b), common bean (Schmutz et al. 2014) and groundnut (Bertioli et al. 2019; Zhuang et al. 2019).

Soybean is a well-studied crop compared to other legumes, and therefore several studies on fine mapping and QTL cloning have been conducted in soybean. For instance, three candidate genes for root knot nematode resistance were identified by using GBS on a RIL population (Xu et al. 2013). NILs were used for map-based cloning of flowering and maturity gene (Watanabe et al. 2011; Tardivel et al. 2014). Two genomic loci responsible for phytophthora resistance, namely *RpsUN1* and *RpsUN2*, were fine-mapped into 151 and 36 kb regions, respectively (Li et al. 2016b). QTL-Seq approach identified a novel *Phytophthora sojae* resistance gene *RpsHC18*, revealing its precise location on chromosome 3 in soybean (Zhong et al. 2018). Positional cloning in soybean with informative recombinants at the *Rhg4* locus enabled Liu et al. (2012a, b) to define an 8 kb region on chromosome 8 controlling resistance to soybean cyst nematode (SCN). Confirmatory evidence for the causative gene underlying *Rhg4* locus, i.e., *serine hydroxymethyltransferase (SHMT)*, was provided through mutant screens, gene expression and gene silencing experiments. More recent use of WGRS data of 106 soybean accessions by the same group has established major role of CNVs in *rhg1* (GmSNAP18) and *Rhg4* (*GmSHMT08*) loci in combination with epistasis and promoter variation for broad-based resistance against SCN (Patil et al. 2019).

In chickpea, skim sequencing has been done on one RIL population to refine the QTL region controlling drought component traits (Kale et al. 2015). This study resolved a broad 7.74 Mb QTL region into ~ 300 kb short segment containing 26 genes. Furthermore, QTL-Seq combined with WGRS identified candidate genes for 100-seed weight (100SDW) and root traits in chickpea (Singh et al. 2016a). These QTLs were co-mapped with the earlier identified QTLs for 100SDW and root traits. Another QTL-Seq study in chickpea identified QTLs for Ascochyta blight on five chromosomes (Ca1, Ca2, Ca4, Ca6 and Ca7) among which QTLs on Ca1, Ca4, Ca6 and Ca7 were overlapped with the earlier identified QTLs using conventional QTL mapping (Deokar et al. 2019). In pigeonpea, marker densities of the genetic maps have been improved incredibly with the recent adoption of NGS techniques (Saxena et al. 2017). Consequently, QTL analysis using high-density genetic linkage maps led authors to detect QTLs in shorter genomic region for disease response (fusarium wilt, sterility mosaic disease: Saxena et al. 2017) and flower, seed-related traits (Yadav et al. 2019). QTL-Seq approach has been used for fine mapping of various important traits in groundnut. Based on the non-synonymous SNPs found between the extreme bulks, allele-specific diagnostic markers were reported for three SNPs for rust and one SNP for LLS (Pandey et al. 2017b). More recently, QTL-Seq by Zhao et al. (2019) localizes *AhTc1* gene in peanut controlling purple testa to a 4.7 Mb region and the underlying J3K16L gene was confirmed through bulked segregant RNA sequencing (BSR-seq) and gene overexpression analyses. A similar QTL-Seq approach in groundnut was associated with 2.4 Mb and 0.74 Mb genomic regions on the pseudomolecules B05 and A09, respectively, with fresh seed dormancy trait (Kumar et al. 2019).

### Is map-based cloning still relevant in the post-NGS era?

In the past two decades, map-based cloning of QTLs/loci for agronomic traits was very popular and several laboratories around the world accomplished fine mapping and cloning of genes by investing > 10 years or so (Salvi and Tuberosa 2005, 2007). One of the main reasons for this included availability of limited markers and requirement of higher costs on sequencing technologies. Identification of millions of SNPs for genetic mapping experiments has now become a common phenomenon. Similarly, new methods of sequencing have brought the costs on sequencing dramatically low. These advances in our opinion have provided a radical change and great opportunity in the way of creating experimental designs and genetic mapping procedures.

For fine mapping in the past, the coarse mapping-based information was used for refining the underlying genomic region by bringing more and more markers. Subsequently, when the QTL region was used to be delimited to very small region on genetic maps, the markers from these regions were used to be deployed for screening large-insert libraries developed using YAC or BAC clones. After identification of positive BAC clones, Sanger sequencing of those clones used provides sequencing of those regions, then predicts the genes and finally shortlists and validates those genes responsible for QTL. However, new ways were introduced in the post-NGS era to avoid these cumbersome procedures. Massive discovery of genome-wide genetic markers like SNPs facilitates quick development of high-density genome maps. QTL mapping using these high-density maps can refine the preliminary QTL regions into candidate gene identification in a faster manner. For instance, if we need to place a QTL into as finer as 10 kb, in case of Arabidopsis we may need ~ 12,500 well-placed markers and in case of papaya ~ 37,200, for rice ~ 43,000 markers, for grape ~ 50,500, for chickpea ~ 73,000, and in the case of large genomes like maize, we may need ~ 2,30,000 and for soybean ~ 1,11,500. Discovery and mapping of such a huge number of genetic markers is now possible by WGRS of entire population (Peters et al. 2003).

The WGRS-based strategy permits placement of a QTL in a genomic region as fine as 10 kb or even lesser. Comparison of those genomic regions with the genome assemblies (as for majority of crops, reference genomes have become available now) can easily identify the well-annotated genes in those regions. In fact, rapid trait mapping approaches such as QTL-Seq can identify the well-annotated genes directly on genome assembly. Such methodological leaps in our opinion indicate non-requirement of traditional cloning methods that seek initial coarse mapping of the genomic region followed by fine mapping to reach candidate loci. The recent post-NGS technologies definitely can help to reduce the time of QTL cloning, and more importantly, one can even bypass the standard fine mapping processes as the primary mapping itself could pinpoint the genes in the QTL region with great precision.

## Conclusion

Fine mapping and QTL cloning were instrumental in understanding the functional mechanism of important plant phenotypes in the past decade. However, sequencing technologies have revolutionized genomics and breeding research in the last decade than in the last 150 years (https://www.lifetechnologies.com/in/en/home/life-science/agricultural-biotechnology/discovery-of-high-density-molecular-markers.html). The resources and time invested to attain candidate genes have been reduced tremendously with these NGS technologies. In the recent past, fine mapping of QTL has become less complicated and rapid due to a variety of reasons: (i) availability of reference genomes for majority of crop species, (ii) availability of high-resolution mapping populations such as MAGIC and NAM, (ii) possibility of construction of genetic maps and undertaking QTL analysis with high marker densities, (iii) possibility of landing directly to genes in QTL regions using rapid trait mapping approaches such as QTL-seq and RenSeq, (iv) availability of gene annotation information for majority of crops to easily shortlist possible genes from the candidate gene lists and (v) availability of mutant populations in several crops and possibility of using fast gene editing approaches for validating gene function. The projects that had taken considerable time (10–15 years) to fine-map, clone QTL and identify a candidate gene now can be completed within a time of 10–20 months.
